# Which Analysis Approach Is Adequate to Leverage Clinical Microdialysis Data? A Quantitative Comparison to Investigate Exposure and Response Exemplified by Levofloxacin

**DOI:** 10.1007/s11095-021-02994-1

**Published:** 2021-03-15

**Authors:** David Busse, André Schaeftlein, Alexander Solms, Luis Ilia, Robin Michelet, Markus Zeitlinger, Wilhelm Huisinga, Charlotte Kloft

**Affiliations:** 1 Institute of Pharmacy, Department of Clinical Pharmacy and Biochemistry, Freie Universitaet Berlin, Berlin, Germany; 2 Graduate Research Training program PharMetrX, Berlin/Potsdam, Germany; 3 Havelland Kliniken GmbH, Hospital Pharmacy, Nauen, Germany; 4 Institute of Mathematics, University of Potsdam, Potsdam, Germany; 5 Clinical Pharmacometrics, Bayer AG, Berlin, Germany; 6 Department of Clinical Pharmacology, Medical University of Vienna, Vienna, Austria

**Keywords:** levofloxacin, microdialysis, noncompartmental analysis, nonlinear mixed-effects modelling, probability of target-attainment

## Abstract

**Purpose:**

Systematic comparison of analysis methods of clinical microdialysis data for impact on target-site drug exposure and response.

**Methods:**

39 individuals received a 500 mg levofloxacin short-term infusion followed by 24-h dense sampling in plasma and microdialysate collection in interstitial space fluid (ISF). ISF concentrations were leveraged using non-compartmental (NCA) and compartmental analysis (CA) via (ii) relative recovery correction at midpoint of the collection interval (midpoint-NCA, midpoint-CA) and (ii) dialysate-based integrals of time (integral-CA). Exposure and adequacy of community-acquired pneumonia (CAP) therapy via pharmacokinetic/pharmacodynamic target-attainment (PTA) analysis were compared between approaches.

**Results:**

Individual AUC_ISF_ estimates strongly varied for midpoint-NCA and midpoint-CA (≥52.3%CV) versus integral-CA (≤32.9%CV) owing to separation of variability in PK parameters (midpoint-CA = 46.5%–143%CV_PK_, integral-CA = 26.4%–72.6%CV_PK_) from recovery-related variability only in integral-CA (41.0%–50.3%CV_recovery_). This also led to increased variability of AUC_plasma_ for midpoint-CA (56.0%CV) versus midpoint-NCA and integral-CA (≤33.0%CV), and inaccuracy of predictive model performance of midpoint-CA in plasma (visual predictive check). PTA analysis translated into 33% of evaluated patient cases being at risk of incorrectly rejecting recommended dosing regimens at CAP-related epidemiological cut-off values.

**Conclusions:**

Integral-CA proved most appropriate to characterise clinical pharmacokinetics- and microdialysis-related variability. Employing this knowledge will improve the understanding of drug target-site PK for therapeutic decision-making.

**Supplementary Information:**

The online version contains supplementary material available at 10.1007/s11095-021-02994-1.

## INTRODUCTION

Clinical pharmacokinetic (PK) studies typically determine drug concentrations in plasma, although this is rarely the target site of the drug. In bacterial infections, the pathogens are mainly found in the interstitial space fluid (ISF), i.e. representing the target site for antibiotics [1]. For several antibiotics, considerable PK differences have been observed between plasma and interstitial fluid [2, 3]. Consequently, the recent European Medical Agency guideline for the development of new antibacterial drugs recommended sampling at the target site [4], mentioning the use of microdialysis, a minimally invasive sampling technique that allows continuous measurements of drug concentrations in the ISF over time [5].

In short, the microdialysis system consists of a catheter with a semipermeable membrane connected to a microdialysis pump (inlet) and to a microvial (outlet) via tubings for dialysate sampling over a collection interval [6]. During microdialysis investigations, perfusate is pumped through the catheter inserted in the ISF of the tissue of interest. The semipermeable membrane at the tip of the catheter allows bidirectional flow of unbound drug molecules along the concentration gradient. After drug intake by a patient, unbound drug molecules present in ISF permeate into the catheter and are transported into the microvial. Due to the constant flow, the drug concentration in the microdialysate will be lower than in ISF. Hence, the measured microdialysate concentration has to be converted by a transformation factor, the relative recovery (RR), obtained using catheter calibration methods. Here, the method of choice in humans is retrodialysis [7], in which retroperfusate is spiked with the drug of interest. RR for the loss of drug from the retroperfusate in the catheter via the membrane into ISF is calculated via Eq. 1:

1 $$ \mathrm{RR}=1-\frac{{\mathrm{C}}_{\mathrm{retrodialysate}}}{{\mathrm{C}}_{\mathrm{retroperfusate}}} $$

with C_retrodialysate_ and C_retroperfusate_ representing retrodialysate and retroperfusate concentration, respectively.

Assuming equal permeation processes in both directions, RR can be used to convert the measured dialysate concentration into ISF concentration, by dividing the microdialysate concentration by RR.

Clinical microdialysis data are commonly evaluated via dialysate-corrected mid-interval based noncompartmental analysis (midpoint-NCA, in the following termed NCA) [8–10] or via nonlinear mixed-effects (NLME) modelling employing dialysate-corrected mid-interval compartmental analysis (midpoint-CA) [11–13]. Both approaches correct the microdialysate concentrations by RR prior to data analysis. This has two important limitations: Data transformation prior to analysis results in loss of information on variability associated with RR and microdialysate concentrations are allocated to a specific time point despite originating from a time interval.

To overcome these limitations, Tunblad et al. developed a dialysate-based integral NLME compartmental analysis (Integral-CA) approach for microdialysis data of morphine/probenecid administration in a small number of rats [14]. Integral-CA integrates all collected drug concentration data (i.e. -if collected- plasma concentrations, retroperfusate, retrodialysate, and microdialysate) simultaneously in one comprehensive model-based analysis. Despite these theoretical benefits of integral-CA over NCA and midpoint-CA, no difference in selected model structure or PK parameter estimates could be shown [14]. Consequently it remains difficult to justify the selection of the more complex and computationally expensive integral-CA over midpoint-CA or NCA for the analysis of clinical microdialysis data. Despite this knowledge gap, recently integral-CA has been regularly employed in analyses of clinical microdialysis data alongside NCA and midpoint-CA [2, 15, 16]. Thus, a quantitative comparison of NCA, midpoint-CA and integral-CA based on a compound with well-characterised PK and availability of clinical target-site concentration data is required.

Levofloxacin, a broad-spectrum fluoroquinolone, encompassing both Gram-negative and Gram-positive organisms including anaerobic bacteria [17], represents such a clinically well-characterised drug, which in addition showed no binding to microdialysis tubing or probes in vitro [18]. It is used for the treatment of various infections, such as community-acquired pneumonia (CAP) [19–21], skin and soft tissue infections, urinary tract infections, and acute exacerbation of chronic bronchitis and sinusitis [22]. Levofloxacin is a moderately lipophilic drug and hence exerts its antibiotic activity in bacteria typically residing in the ISF. It has been shown that the most relevant predictor of fluoroquinolone efficacy in clinical settings is the 24-h area under the unbound drug concentration-time curve (*f*AUC_0–24_) divided by the minimum inhibitory concentration (MIC) [23] for different bacteria strains.

The objectives of this study were to quantitatively compare (1) levofloxacin exposure calculated via NCA, midpoint-CA and integral-CA in plasma and at target site, and (2) the PK/pharmacodynamic (PD) evaluation of current dosing regimens evaluated via midpoint-CA and integral-CA. Ultimately, the aim was to derive recommendations for target-site data analysis integrating all available measurements obtained in microdialysis studies.

## METHODS

Model comparison was based on PK measurements of four single centre, clinical microdialysis studies in patients with soft tissue infections (*n* = 8) [24], patients receiving coronary bypass surgery (*n* = 12) [10], patients undergoing elective lung surgery (*n* = 5) [25] as well as healthy volunteers (*n* = 7) and septic patients (n = 7, Table I) [26]. All individuals received 500 mg levofloxacin (Tavanic®; SANOFI, Frankfurt/Main, Germany) administered as a single intravenous infusion over 30 min, except for the lung surgery patients, who received a 1-h infusion of the same dose. Blood samples and microdialysis data (20–120 min collection intervals, with ≤30 min intervals for the first 2 h after drug administration and ≥60 min intervals for the rest of the collection time) were collected up to 24 h after the start of the infusion [10, 24–26]. In all participants, microdialysis catheters were inserted into the ISF of subcutaneous adipose tissue of the thigh and skeletal muscle of the thigh and perfused with Ringer’s solution. Additionally, in soft tissue infection patients, catheters were placed in ISF of infected and noninfected subcutaneous adipose tissue. Retrodialyses were carried out for 30 min [10, 24–26], yielding a single sample per catheter. Plasma, microdialysis and retrodialysis samples were analysed by a validated HPLC assay [27].

**Table I:** Baseline characteristics in the study populations

Population	Number of patients	Percentage of patients
Soft tissue infection patients	8	20.5%
Coronary bypass surgery patients	12	30.8%
Lung surgery patients	5	12.8%
Healthy volunteers	7	17.9%
Septic patients	7	17.9%
Continuous patient characteristics	Median	5^th^ – 95^th^ percentile
Age [y]	61	23–89
Weight [kg]	75	51–120
Height [m]	1.70	1.54–1.87
Serum albumin concentration [g/l]	22.4	18.0–57.0
Creatinine clearance [ml/min]*	82.1	37.3–146
C-reactive protein concentration [mg/l]	6.30	0.500–21.3
Leucocyte concentration [10^9^ cells/l]	7.35	5.06–14.3
Categorical patient characteristics	Number of patients	Percentage of patients
Sex (male)	25	78.1%

*calculated via Cockcroft Gault formula

### Noncompartmental Analysis

NCA was performed in PKanalix (Monolix Suite 2019R1, Lixoft, France). The linear-up, log-down approach was employed with uniform weighing for log-linear regression of the terminal phase, which was evaluated via standard goodness-of-fit plots (e.g. observed versus predicted dependent variables, weighted residuals versus prediction/time). Extrapolations were based on predicted last concentrations. The impact of individual characteristics on PK parameters obtained via NCA was evaluated by linear regression in R.

### Nonlinear Mixed-Effects Modelling Analysis

In the NLME analysis via integral-CA and midpoint-CA, candidate PK models with plasma data attributed to the central compartment and target-site data attributed to central or peripheral compartments were evaluated in the NLME modelling software NONMEM v7.4.3 (Icon Development Solutions, Ellicott City, MD, USA) accessed with PsN v4.8.1 [28] through Pirana v2.9.6 (Certara, Princeton, NJ, USA). R v3.6.0 (R Foundation for Statistical Computing, Vienna, Austria) was used for dataset preparation, model evaluation and post-processing results. Intercompartmental flows and elimination from the central compartment were assessed using linear and nonlinear processes.

The following clinical and demographic characteristics were considered biologically plausible to affect levofloxacin PK and were tested for inclusion as continuous covariates on selected model parameters: Age, creatinine clearance (CLCR) [29], leukocyte blood concentration, C-reactive protein concentration, gamma-glutamyl transferase, alanine and aspartate aminotransferase, serum albumin concentration, as well as body height and weight including derived alternative body size descriptors such as body mass index and lean body weight determined according to [30]. Additionally, the impact of the categorical covariates sex and disease type was tested.

Statistical comparisons between nested models with additional covariates were made using the likelihood ratio test [31]. Model selection was further based on statistical significance (*p*<0.05), plausibility and precision of parameter estimates. NLME models were evaluated by standard goodness-of-fit plots. The predictive model performance was assessed by visual predictive checks (*n* = 1000); the uncertainty, bias and robustness of parameter estimates were evaluated by nonparametric bootstrap (n = 1000) [32]. A detailed description of considered model equations can be found in the Supplementary information. Accordance in exposure estimates obtained via NLME approaches and NCA was evaluated by linear regression in R. To evaluate the extent of distribution into the target site, penetration indices were calculated as *f*AUC_ISF_/*f*AUC_plasma_ over time for adipose and muscle tissue.

### Probability of Target Attainment and Cumulative Fraction of Response Analysis

Due to the usage of levofloxacin in the treatment of lung infections, such as CAP [19, 20], the adequacy of levofloxacin dosing regimens recommended for the treatment of CAP [21] was evaluated by probability of target attainment (PTA) analysis, a pharmacometric tool aimed at optimising dosing regimens of anti-infectives with the goal of improving therapeutic outcome and preventing the emergence and spread of resistance [33]. For this purpose, Monte Carlo simulations (*n* = 1000 per covariate combination) of levofloxacin concentration over time in plasma were performed for the integral-CA and midpoint-CA models for different dosing regimens. To cover a broad range of PK/PD targets and MIC values reported for levofloxacin by the European Committee on Antimicrobial Susceptibility Testing (EUCAST), PTA for achieving the CAP-related PK/PD target *f*AUC_0–24_/MIC≥34 [34] was calculated for MIC = 0.100–4.00 mg/l [35]. A plasma protein binding of 31.0%, the average across reported values, was selected for calculation of unbound levofloxacin concentrations [36–39]. PTA was calculated using the percentage of the simulated individuals who were at or above the PK/PD target at each MIC. A dosing regimen was considered adequate if PTA≥90%.

The adequacy of levofloxacin dosing regimens was additionally evaluated against the most prevalent CAP-associated pathogens (*Haemophilus influenzae, Staphylococcus aureus and Streptococcus pneumoniae)* [35, 40]. For this purpose, the cumulative fraction of response (CFR) according to [41] was calculated by multiplying PTA by the cumulative proportion of bacterial isolates at each MIC from EUCAST (MIC = 0.001–512 mg/l).

To consider relevant dosing regimens for the treatment of CAP, once- and twice-daily intravenous short-term (30 min) infusions of 500 mg levofloxacin were evaluated [21].

### Comparison of Analysis Approaches

To identify the most appropriate analysis approach for the evaluation of microdialysis data the competing analysis approaches were compared based on (i) precision (only NLME modelling approaches) and plausibility of parameter estimates and (ii) goodness-of-fit and predictive model performance. Subsequently, the impact of the choice of the analysis approach on (a) levofloxacin exposure and (b) PK/PD evaluation of levofloxacin dosing regimens (only NLME modelling approaches) was evaluated. As the applied approaches to evaluate PTA and CFR relied on estimates of interindividual variability through Monte Carlo simulation, only integral-CA and midpoint-CA, but not NCA, were compared.

## RESULTS

**Fig. 1:**
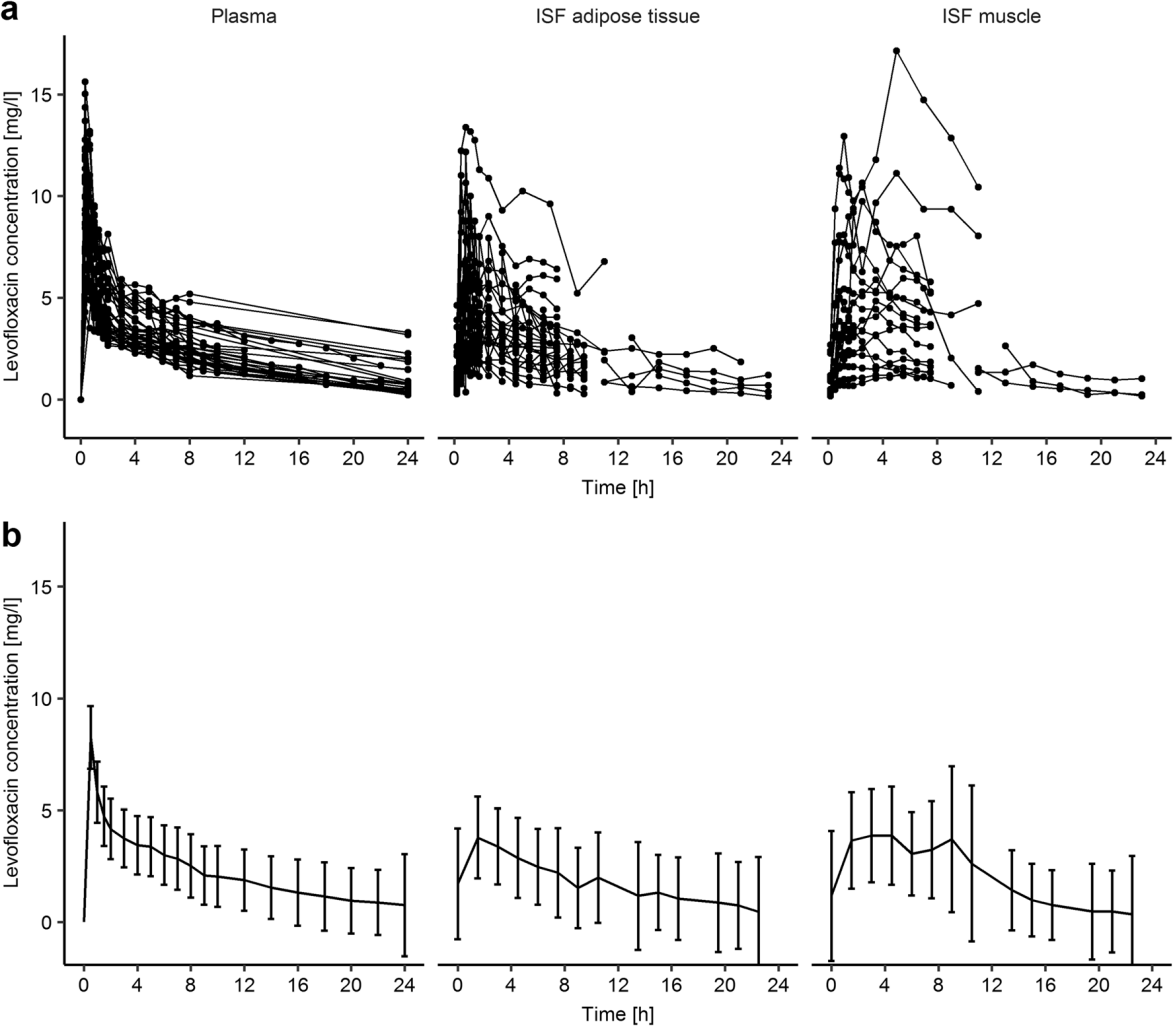
Individual observations (**a**) and geometric mean and geometric standard deviation (**b**) of levofloxacin concentrations over time in plasma (total) and ISF (interstitial space fluid) of adipose tissue and muscle (both unbound)

Per individual on average (range) 13 plasma concentrations (3–15, *n* = 39 individuals), 14 microdialysate collections in ISF of adipose tissue (6–28, *n* = 28 individuals) and 11 in ISF of muscle (6–12, *n* = 24 individuals) were obtained after the start of the levofloxacin administration. The geometric mean and 10^th^–90^th^ percentiles of the maximum levofloxacin concentration (C_max_) in plasma were 8.25 mg/l, 7.04–13.5 mg/l (Fig. 1b), but observations at the end of the infusion were only available for lung surgery patients (*n* = 5, 12.8% of all patients). The geometric mean of C_max_ in ISF of adipose tissue (4.65 mg/l, 7.50–23.0 mg/l) and muscle (4.83 mg/l, 1.52–11.1 mg/l) were comparable and delayed in comparison to plasma (t_max_ = 1.64 h, 0.796–11.4 h, and t_max_ = 3.48 h, 1.17–11.0 h, respectively).

### Precision and Plausibility of Parameter Estimates

**Fig. 2:**
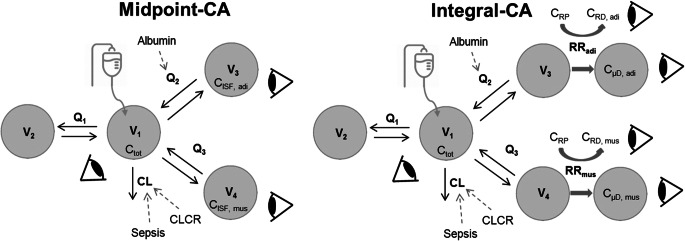
Schematic levofloxacin nonlinear mixed-effects models via midpoint-CA (dialysate-corrected mid-interval compartmental analysis; left) and integral-CA model (dialysate-based integral compartmental analysis; right). Solid arrows indicate mass transfer, grey dotted arrows indicate covariate relationships and bold grey arrows indicate input and scaling from the retrodialysis submodel. Eyes denote observations included in the model. Adi: adipose tissue, CA: compartmental analysis, CL: clearance, CLCR: creatinine clearance, C_ISF_: calculated interstitial space fluid concentration, C_RD_: retrodialysate concentration, C_RP_: retroperfusate concentration, C_tot_: total plasma concentration, C_μD_: unbound microdialysate concentration, mus: muscle, Q: intercompartmental flow, RR: relative recovery, V: volume of distribution

While the NCA approach did not necessitate structural model development, the same final PK model structure was identified for integral-CA and midpoint-CA: A mammillary four-compartment model with clearance from the central compartment (Fig. 2). Plasma concentrations were related to the central compartment and target-site concentrations were attributed to distinct peripheral compartments.

Total volume of distribution (V_tot_) differed between NCA (V_tot_,_mean_ = 104 l, at steady-state) and population estimates from the NLME approaches midpoint-CA (V_tot_ = 82.7 l) and integral-CA (V_tot_ = 81.1 l, Table II). Both population estimates of V_tot_ were in accordance with the NLME model based on plasma concentrations only (V_tot_ = 86 l, Tab. AI). However, compared to the central volume of distribution (V_1_) in integral-CA and the plasma only model (15.5 l and 21.7 l, respectively), V_1_ was largely decreased in midpoint-CA (4.82 l, Tab. AII). Clearance obtained from all three approaches (7.35–8.26 l/h) was comparable.

**Table II:** Parameter estimates from the noncompartmental analysis (NCA), the dialysate-corrected mid-interval compartmental analysis (midpoint-CA) and the dialysate-based integral compartmental analysis (integral-CA)

	**NCA**		**Midpoint-CA**	**Integral-CA**
Structural model	–		4 compartment mammillary	4 compartment mammillary
**Structural PK parameters**				
V_tot_ [l]	104		82.7	81.1
CL [l/h]	8.26		7.35	7.78
Q_tot_ [l/h]	–		115	97.6
Adipose tissue k_13_/k_31_	–		1.51	2.14
Muscle k_14_/k_41_	–		4.44	1.03
**Impact influential patient characteristics**				
CL-CLCR [(ml/min)^−1^] ^a^	0.90%^1^		1.26%^2^	1.12%^2^
CL_sepsis_^b^	−45.5%^1^		−52.8%^2^	−43.4%^2^
Q_adi_-albumin [(g/l)^-1^] ^c^	–		8.19%^2^	7.25%^2^
**Stochastic parameters**				
IIV (range)	–		46.5%CV-143%CV	26.4%CV-72.6%CV
RR related variability (adipose/muscle)	–		–	40.1%CV/50.3%CV
Residual variability	–		30.3%CV^3^	8.83%CV-28.3%CV^4^

^1^Estimated via linear regression of CL versus CLCR centred on median and separate intercepts for sepsis patients and patients without sepsis. ^2^Estimated via linear/categorical parameter-covariate relationships. ^3^Single residual variability term for all biological matrices. ^4^Separate residual variability terms for all biological matrices. ^a^Change of clearance per ml/min deviation of CLCR from 82.1 ml/min. ^b^Change of clearance in patients with sepsis compared to patients without sepsis. ^c^Change of Q_adi_ per g/l deviation of serum albumin concentration from 22.4 g/l.

CL: clearance, CLCR: creatinine clearance, CV: coefficient of variation, IIV: interindividual variability, k_13_: transfer rate constant from central to adipose interstitial space fluid compartment, k_14_: rate constant from central to muscle compartment, k_31_: rate constant from adipose tissue compartment to central compartment, k_41_: rate constant from muscle compartment to central compartment, Q_tot_: sum of intercompartmental flows, Q_adi_: intercompartmental flow between central compartment and adipose tissue compartment, V_tot_: total volume of distribution, RR: relative recovery

Volumes of distribution associated with ISF of adipose tissue and muscle and the sum of intercompartmental flows were comparable between midpoint-CA and integral-CA (Table II). In contrast, ratios of rate constants into and from the target site were similar for ISF of adipose tissue (1.51 for midpoint-CA and 2.14 for integral-CA) but differed largely for muscle (4.44 for midpoint-CA and 1.03 for integral-CA).

Identical relationships between PK parameters and patient characteristics were identified for the three approaches: Clearance increased by 9.00%–12.6% for each 10 ml/min increase of CLCR (Table II), and was reduced by 43.4%–52.8% in sepsis patients. The intercompartmental flow (not available for NCA due to the lack of mass transfer descriptions) associated with ISF of adipose tissue increased by 7.25%–8.19% for each g/l increase in serum albumin concentration. The inclusion of these covariate relationships decreased the overall interindividual variability in the NLME models substantially (relative reduction of coefficient of variation ≤30.1% for midpoint-CA and ≤43.7% for integral-CA).

On average, estimates of interindividual variability (not available for NCA) were higher in midpoint-CA versus integral-CA (Δ%CV_mean_ = 27.1%). Only integral-CA allowed discrimination of residual variability attributed to microdialysis, retrodialysis and plasma measurements (8.83%–28.3%CV, Tab. AII). Here, residual variability on retrodialysis (18.4%–24.9%CV) and microdialysis (25.4%–28.3%CV) was higher than for plasma observations (8.83%CV).

Parameter precision of the structural parameter estimates was comparable between midpoint-CA (relative standard error (RSE): 10.9%–48.3%) and integral-CA (RSE: 3.89%–61.7%) but parameter precision of interindividual variability of the key PK parameters clearance and V_1_ was lower for midpoint-CA (RSE: 62.5% and 71.2%, respectively) versus integral-CA (RSE: 38.3% and 34.2%, respectively; Tab. AII), highlighting an important advantage of integral-CA (Table III).

**Table III:** Summary of performance evaluation of noncompartmental analysis (NCA), dialysate-corrected mid-interval compartmental analysis (midpoint-CA) and the dialysate-based integral compartmental analysis (integral-CA)

**Evaluation criteria**	**NCA**	**Midpoint-CA**	**Integral-CA**
Computational time^1^	short (<2.00 s)	medium (1150 s)	long (24000 s)
Precision of PK parameter estimates^2^	n.a.	–	+
Goodness-of-fit^3^	(+)*	–	+
Predictive PK model performance^4^	n.a.	–	+
Differentiation between microdialysis technique- and PK-related variability	–	–	+
Plausibility of penetration indices (*f*AUC_ISF_/*f*AUC_plasma_)	–	+	+

^1^CPU time on a 360 GHz Intel Core i7–7700

^2^Relative standard error of key pharmacokinetic parameters from bootstrap <50%

^3^Visual inspection of trends in standard goodness-of-fit plots

^4^Assessed via visual predictive checks

*Limited to terminal phase of levofloxacin concentration-time profile

-: inadequate result/unapt; +: optimal result/apt; *f*AUC: area under the unbound concentration-time curve; ISF: interstitial space fluid; n.a.: not applicable; PK: pharmacokinetic

### Goodness-of-Fit and Predictive Performance of Nonlinear Mixed-Effects Modelling Approaches

In contrast to midpoint-CA, which demonstrated trends indicating model overpredictions (plasma and adipose tissue) and underpredictions (muscle) in the middle of the measured concentration range (Fig. A1), no pronounced trends were evident for integral-CA (Fig. A2), showing superiority of integral-CA (Table III).

After accounting for interindividual differences in RR, remaining differences between predicted retrodialysate concentrations (used in integral-CA model to calculate RR) and observed ones (used in midpoint-CA and NCA to calculate RR) were evident (Fig. A2b2 and b4). This divergence was quantified as residual unexplained variability (Table AII).

Whereas all data points were evaluated in the NLME approaches, NCA predictions were limited to the terminal phase of the levofloxacin concentration-time profile in all matrices employed for log-linear regression (Fig. A3).

**Fig. 3:**
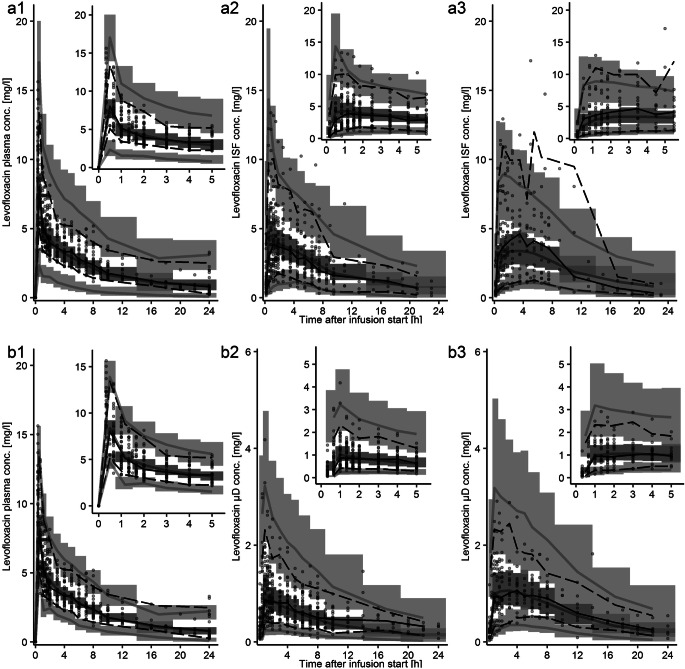
Visual predictive check (*n* = 1000 simulations) for the dialysate-corrected mid-interval model “(midpoint-CA)” (**a**) and the dialysate-based integral compartmental analysis “(integral-CA)” (**b**) for total plasma concentrations (1), microdialysate concentrations for ISF in adipose tissue (2) and ISF in muscle (3). Circles: Observed levofloxacin concentrations; Lines: 5^th^, 95^th^ percentile (dashed), 50^th^ percentile (solid) of the observed (black) and simulated (grey) data. Shaded areas: 95% confidence interval around 5^th^, 50^th^ and 95^th^ percentile of simulated data. μD: microdialysate; conc.: concentration; ISF: interstitial space fluid

In the visual predictive check of the integral-CA, the median of the simulated concentration-time profiles corresponded with the median of the observed data of all matrices, whereas the 5^th^ and 95^th^ percentile of simulations were lower and higher, respectively, than observed ones. Yet, observed percentiles lay within the 95% confidence intervals of simulated ones, indicating an overall good predictive model performance (Fig. 3b and Fig. A4). In contrast, for midpoint-CA the 5^th^ and 95^th^ percentile of the simulated concentration-time profile diverged from those of observations for numerous sampling time points in plasma and ISF of muscle (Fig. 3a), indicating over prediction of PK-related variability. In addition, for ISF of muscle the median simulated midpoint-CA profile indicated model misspecifications. The less accurate predictive performance compromised the applicability of midpoint-CA for Monte Carlo simulations (Table III) and therefore the subsequent PK/PD evaluation through PTA analysis.

### Comparison of Levofloxacin Exposure between Approaches

**Fig. 4:**
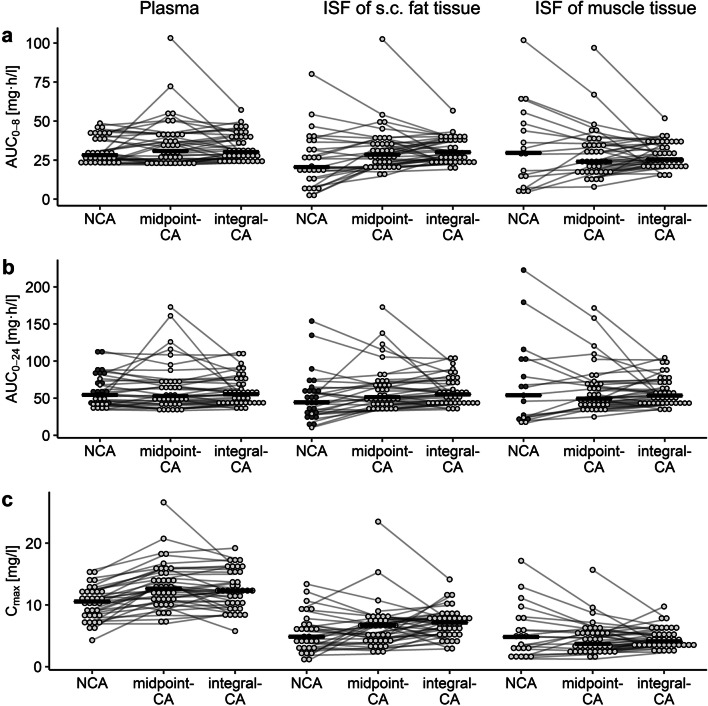
Comparison of individual area under the levofloxacin concentration-time curves from 0 to 8 h (AUC_0–8_) and 0–24 h (AUC_0–24_) and maximum concentration (C_max_) determined via noncompartmental analysis (NCA), dialysate-corrected mid-interval compartmental analysis (midpoint-CA) and dialysate-based integral compartmental analysis (integral-CA). Plasma exposure (left column) is expressed as total and exposure in the interstitial space fluid (ISF) of subcutaneous (s.c.) fat tissue (mid column) and in ISF of muscle tissue (right column) as unbound. Black line represents the median, grey lines connect exposure metrics from the same individual, dark grey dots represent individuals with fraction of AUC extrapolated until infitiy>20%

A comparison of individual exposure ranges determined via NCA, midpoint-CA and integral-CA (Fig. 4a-b) revealed differences in AUC from 0 to 8 h (AUC_0–8_) and AUC_0–24_ in plasma between NCA (AUC_0–8,range_ = 22.0–48.6 mg∙h/l, AUC_0–24,range_ = 34.1–114 mg∙h/l) and integral-CA (AUC_0–8,range_ = 22.8–57.1 mg∙h/l, AUC_0–24,range_ = 34.7–112 mg∙h/l), versus midpoint-CA (AUC_0–8,range_ = 21.7–103 mg∙h/l, AUC_0–24,range_ = 33.1–173 mg∙h/l). This divergence of midpoint-CA was further substantiated by the low concordance (Lin’s concordance correlation coefficient ≤ 0.546) of individual plasma AUC estimates between the midpoint-CA versus integral-CA and NCA (Fig. A5-A6). The median plasma C_max_ (10.6 mg/l) was lower for NCA compared to midpoint-CA (12.7 mg/l) and integral-CA (12.3 mg/l, Fig. A7), since for most individuals sampling times did not include the end of the infusion (hence t_max_ was not covered), highlighting a well-known inherent limitation of NCA versus NLME approaches based on the sampling design.

Variability of individual AUC at target site was larger for NCA (69.1%CV-79.2%CV) and midpoint-CA (52.3%CV-56.0%CV) compared to integral-CA (32.6%CV-32.9%CV, Fig. 4) and the accordance of estimates between the different methods was low (Fig. A5-A7). By design, individual AUC determined via NCA and midpoint-CA in both matrices was dependent on RR, whereas no dependency was evident for integral-CA (*p* = 0.0895 and *p* = 0.265, Fig. A8), highlighting an advantage of integral-CA (Table III). Importantly, NCA measures of AUC had a large proportion (>20% [42]) of AUC extrapolated until infinity in plasma (45.7%), ISF of adipose tissue (23.5%) and ISF of muscle (38.2%).

**Fig. 5:**
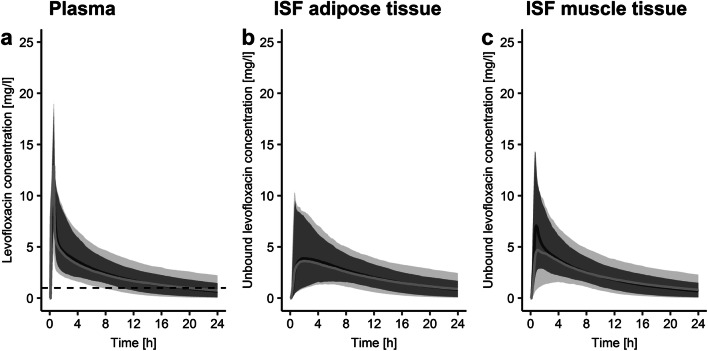
Levofloxacin total plasma concentration and unbound ISF (interstitial space fluid) concentrations over time for the dialysate-corrected mid-interval (light grey) and the dialysate-based integral compartmental analysis (dark grey). Median (solid lines) and 95% CI (shaded bands) from 1000 Monte-Carlo simulations are shown. The non-species related resistance breakpoint in plasma (1 mg/l) is visualised as a dotted line

**Fig. 6:**
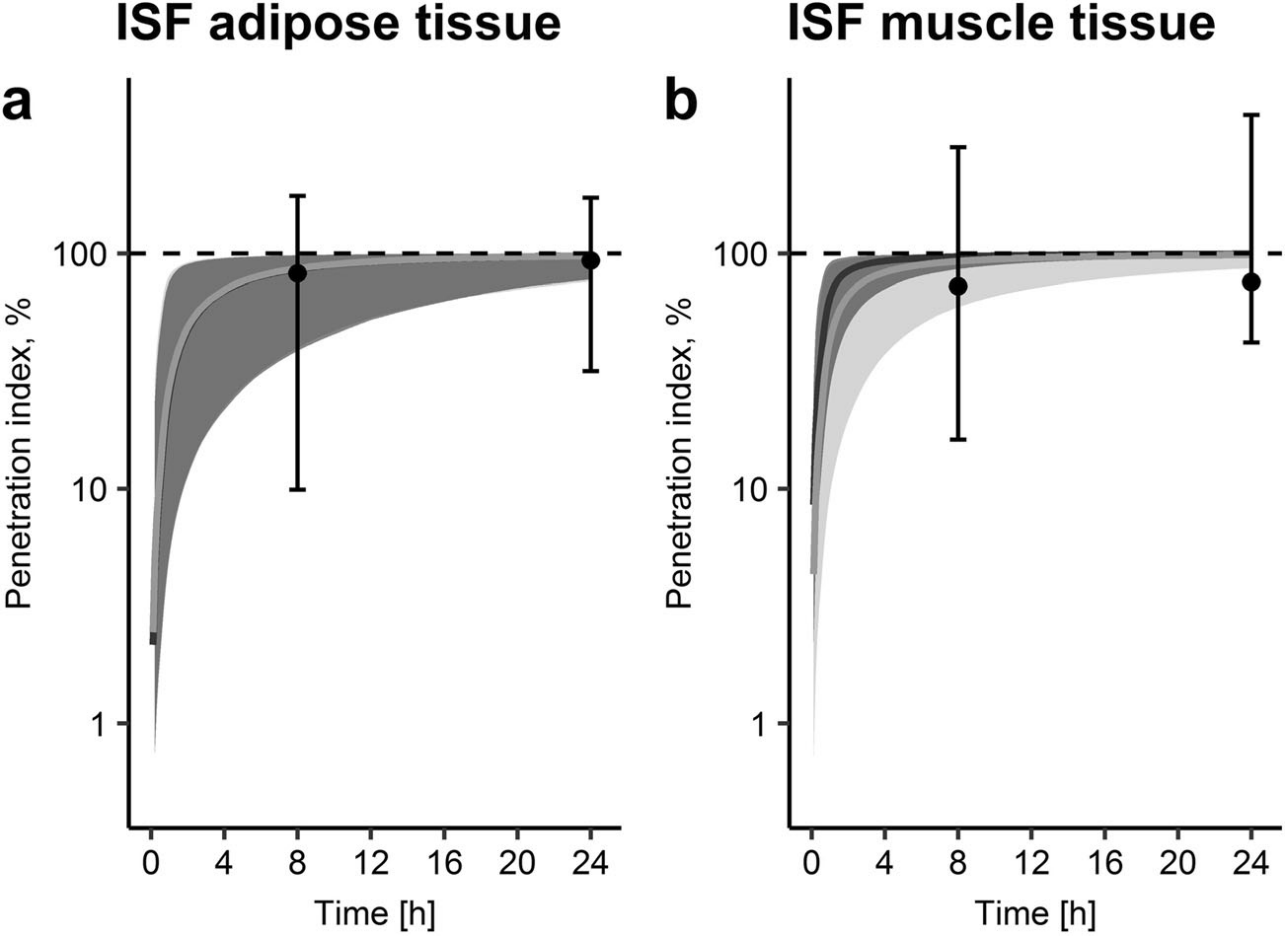
Levofloxacin penetration indices (ratios of unbound area under the concentration-time profiles in plasma and target sites) for ISF (interstitial space fluid) over time for the dialysate-corrected mid-interval (“midpoint-CA”, light grey) and the dialysate-based integral compartmental analysis (“integral-CA”, dark grey). Median (solid lines) and 95% CI (shaded bands) from 1000 Monte-Carlo simulations are shown. Dots and whiskers show median, 2.5^th^ and 97.5^th^ percentiles of penetration indices calculated via non-compartmental analysis

The larger variability associated with exposure simulations from midpoint-CA compared to integral-CA led to broader 95% CI bands obtained from Monte Carlo simulations for all three matrices (Fig. 5). The difference between rate constants associated with ISF of muscle tissue estimated via midpoint-CA and integral-CA manifested in differences in predicted C_max_ (C_max__,median_ = 5.38 mg/l versus 7.49 mg/l, respectively, Fig. 5c). Similarly, penetration indices (AUC_ISF_/AUC_plasma_) were relatively similar for both approaches for ISF of adipose tissue but differed largely for muscle tissue (Fig. 6). Whereas penetration indices obtained from NLME approached 100% towards the end of the sampling time, penetration indices obtained via NCA yielded penetration indices>100% (Fig. 6). The implausibility of penetration indices>100% indicated a further disadvantage of NCA (Table III).

### Pharmacokinetic/Pharmacodynamic Evaluation of Levofloxacin Dosing Regimens Based on Nonlinear Mixed-Effects Modelling Approaches

Monte-Carlo simulations of levofloxacin plasma concentrations following dosing regimens recommended for the treatment of CAP (once- and twice-daily 500 mg levofloxacin short-term infusions of 0.5 h [21]) were performed covering the 5^th^ to 95^th^ percentile of study CLCR (37.3–146 ml/min) of individuals without sepsis.

**Fig. 7:**
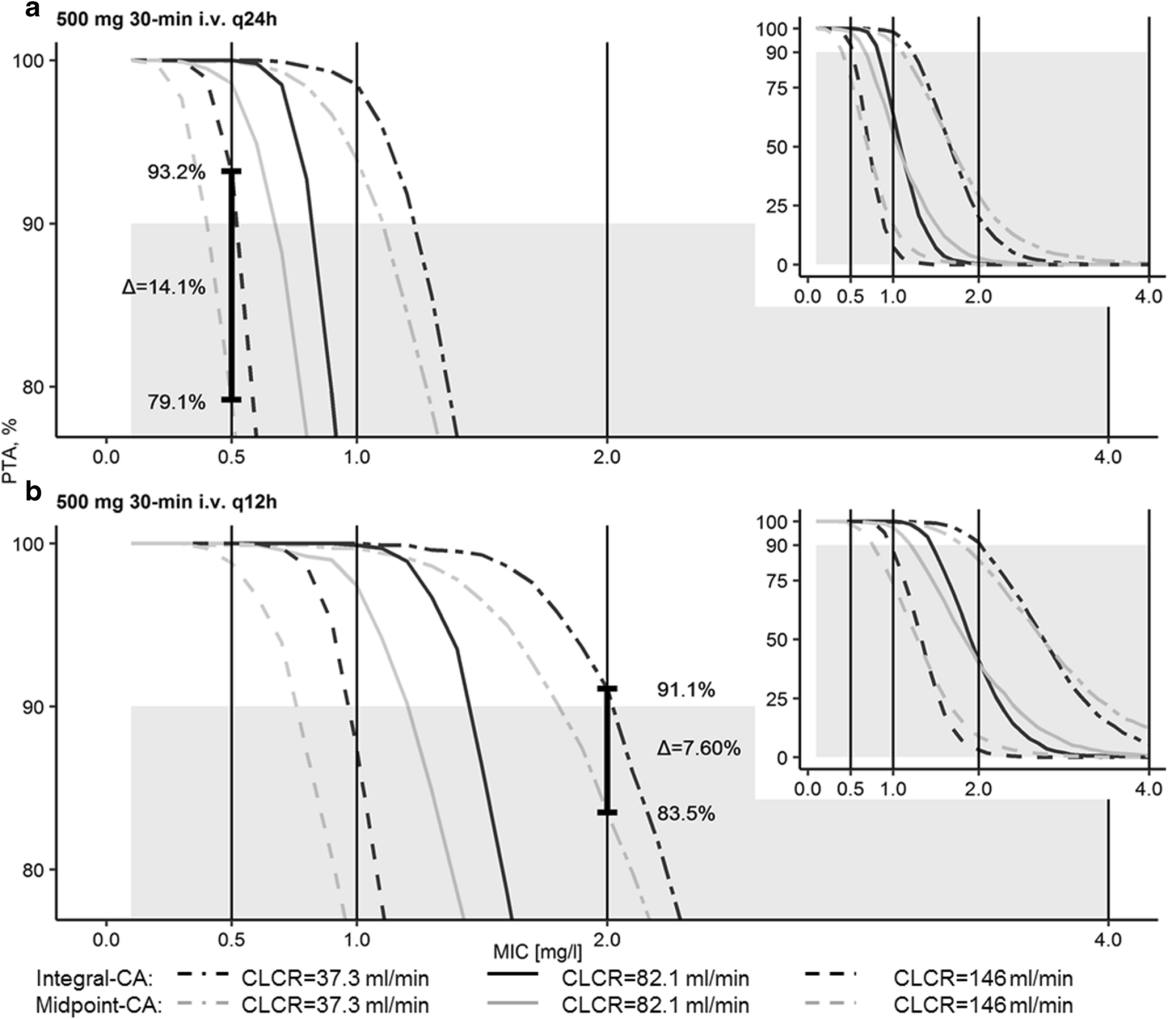
Probability of target attainment (PTA) versus minimum inhibitory concentration (MIC) of dosing regimens for the treatment of community-acquired pneumonia (CAP): 30-min 500 mg levofloxacin intravenous infusions once (q24h, a) and twice daily (q12h, b). PTA<90% (inadequacy of dosing regimen) is shaded in grey. Vertical lines represent MIC values including epidemiological cut-off values of the CAP-related pathogens *Staphylococcus aureus* (MIC = 0.5 mg/l) and *Streptococcus pneumoniae* (MIC = 2.0 mg/l). Differences in the evaluation of adequacy of dosing regimens between the dialysate-based integral compartmental analysis (integral-CA) and dialysate-corrected mid-interval compartmental analysis (midpoint-CA) at each MIC value are denoted as bold vertical lines. Inset plots show complete PTA profiles versus MIC. CLCR: creatinine clearance

PTA was lower at higher CLCR and PTA calculated via integral-CA (PTA_integral-CA_) was higher than PTA determined via midpoint-CA (PTA_midpoint-CA_) when PTA≥90% (relevant PTA range for the evaluation of adequacy of dosing regimens, Fig. 7): For the epidemiological cut-off value (ECOFF) of *Staphylococcus aureus* (MIC = 0.5 mg/l) the administration of 500 mg once-daily to an individual with healthy/elevated renal function (CLCR = 146 ml/min) was only deemed adequate via integral-CA but not midpoint-CA (Fig. 7a). The same applied to the ECOFF of *Streptococcus pneumoniae* (MIC = 2.0 mg/l) and the twice-daily administration of 500 mg levofloxacin to an individual with severe renal impairment (CLCR = 37.3 ml/min, Fig. 7b). At low PTA (⪅50%), midpoint-CA yielded larger PTA than integral-CA.

**Fig. 8:**
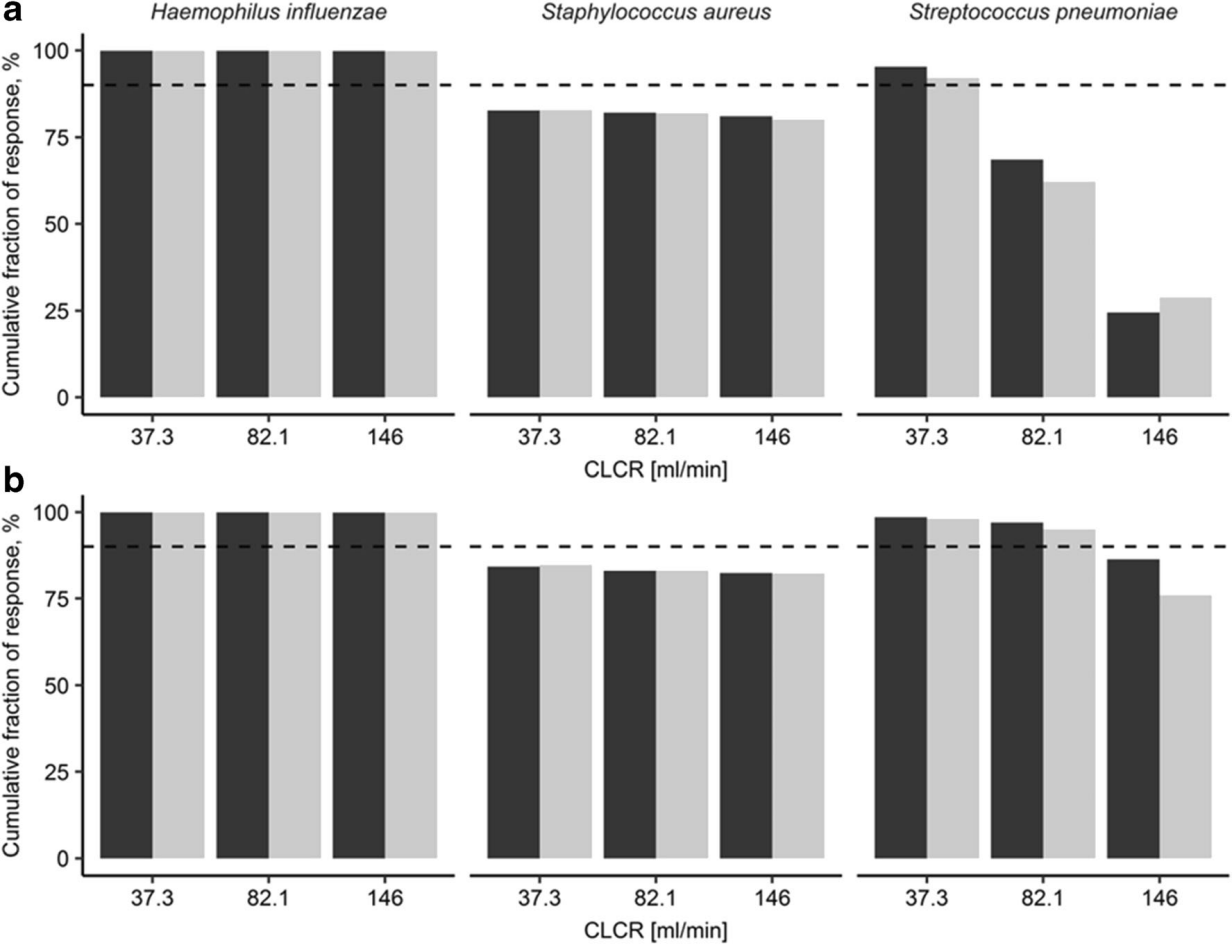
Cumulative fraction of response (CFR) versus creatinine clearance (CLCR) for 30-min 500 mg levofloxacin intravenous infusions once (**a**) and twice (**b**) daily for the most relevant community-acquired pneumonia pathogens. CFR for the dialysate-based integral compartmental analysis is labelled black and CFR for the dialysate-corrected mid-interval compartmental analysis is labelled grey. The dashed line denotes adequate coverage (≥90%)

When considering species-specific MIC distributions via CFR analysis of the most common CAP associated pathogens, both NLME approaches evaluated the once-daily dosing regimen as adequate to cover *Haemophilus influenzae* but not *Staphylococcus aureus* (Fig. 8a). Adequate CFR to *Streptococcus pneumoniae* for all investigated individuals was only achieved by a twice-daily dosing regimen and for CLCR<146 ml/min (Fig. 8b). Integral-CA showed higher CFR than midpoint-CA for *Streptococcus pneumoniae* (up to 10.4% points).

## DISCUSSION

Our study demonstrated inferiority of midpoint-CA and NCA versus integral-CA to analyse clinical microdialysis PK data (Table III), which resulted in inflated variability of exposure estimates. As a result ~1/5 of penetration indices obtained from NCA were >100%, which was implausible since levofloxacin (and other fluoroquinolones) demonstrated no accumulation in the ISF [43]. Secondly, the evaluation of adequacy of both levofloxacin dosing regimens employed for the treatment of CAP for selected ECOFF via midpoint-CA was erroneous in 1/3 patient cases. Thus, the observed pronounced overestimation of variability in levofloxacin PK via midpoint-CA had clinically relevant consequences on the PK/PD evaluation of levofloxacin dosing regimens. Yet, no clinically relevant differences in CFR calculated via the two analysis approaches for CAP-related pathogens were evident.

Compared to midpoint-CA, estimates of interindividual variability of integral-CA were lower, which can be explained by the differentiation of the drug PK processes and microdialysis associated variabilities through incorporation of RR in the model, representing a key advantage of the integral-CA approach. In the integral-CA, interindividual variability of disposition-related parameters was determined independently from variabilities of the microdialysis technique. This allowed more reliable estimates of interindividual variability (as shown in visual predictive checks) and covariate effects due to a reduced propensity of measurement technique-dependent influences. Further illustration was provided by the lack of a dependency of individual AUC estimates on RR.

In contrast to estimates of interindividual variability, levofloxacin plasma PK parameters were relatively similar for the three analysis approaches. V_tot_ (NCA: 1.39 l/kg, midpoint-CA: 1.10 l/kg, integral-CA: 1.08 l/kg) and clearance (NCA: 0.110 l/h/kg, midpoint-CA: 0.0980 l/h/kg, integral-CA: 0.104 l/h/kg) were in accordance with prior literature results (0.800–1.50 l/kg for V_tot_ and 0.120–0.141 l/h/kg for clearance) [39, 44–47], indicating robustness of estimates obtained by either analysis approach.

Despite the general agreement of structural PK parameter estimates between NLME modelling approaches and NCA, the latter only allowed separate evaluation of the three available sources of observations (plasma, ISF in adipose tissue and muscle), instead of accounting for mass transfer of levofloxacin by integrating observations of plasma and target site simultaneously. This prevented the evaluation of the time scale of equilibration of target sites with plasma, led to implausibly high values of penetration indices above 100% at 24 h (24.6%–188% for ISF in adipose tissue and 39.1%–508% for ISF in muscle) and did not allow PK/PD evaluation through Monte Carlo simulations, limiting the general applicability of this approach.

Our findings on clinical relevance of the selected analysis approach represented an expansion to the comparison for preclinical microdialysis data provided by Tunblad et al. [14], who reported results for the morphine model in rats. To demonstrate how exposure differences can translate into altered clinical evaluations of current dosing regimens based on the selected analysis approach, we further included a comparison on the evaluation of dosing regimens via PTA and CFR analysis. Tunblad et al. found only minor differences in interindividual variability in PK parameters of midpoint-CA and integral-CA, likely owing to the low variability of RR in their study, which has been determined in a standardised preclinical animal model under laboratory conditions (14.0%CV) compared to our RR values determined in patients in the clinic (41.0%CV-50.3%CV). With oftentimes large variability of RR reported in the clinic [48, 49] the risk of obtaining inflated estimates is high and thus the choice of a suitable method to analyse microdialysis data is decisive.

PTA and CFR analysis were performed based on plasma exposure since the currently employed PK/PD targets were defined for AUC in plasma [34]. As indicated by levofloxacin penetration indices≈1 at 24 h for both target sites, *f*AUC_0–24_ was similar in plasma and the target sites; hence results of PTA in plasma and at target site would be similar as well. However, the defined PK/PD targets might require revision, especially with more abundant knowledge of drug concentration-time profiles at target site instead of plasma exposure alone.

The midpoint-CA approach relied on attribution of the calculated ISF concentration to the mid of the collection interval length, which might be adequate given linear pharmacokinetics and small changes in the concentrations over time in each collection interval [14]. However, in case of longer collection intervals, fast change of concentrations over time or non-linear pharmacokinetics, larger errors of the time scale are likely and biased parameter estimates might be obtained.

In general, which concentration reflects the “true” concentration in the ISF hitherto cannot be conclusively clarified, especially due to the lack of comparable, alternative measurement methods for unbound concentration in the ISF. This should be further evaluated via simulation-based studies. Such investigations should focus on the impact of sampling interval length inclusion of retrodialysis-related observations in the model, variability in RR and pharmacokinetic drug properties (e.g. time scale of distribution into target site) on the bias of parameter estimates to derive general rules when midpoint-CA and NCA are sufficient or the computationally more expensive integral-CA is required.

In conclusion, although NCA, midpoint-CA and integral-CA yielded similar and plausible structural PK parameter estimates, yet when evaluating interindividual variabilities of PK parameters, only integral-CA allowed adequate model predictivity. This translated into clinically relevant differences in the PK/PD evaluation of recommended dosing regimens of levofloxacin for the treatment of CAP between midpoint-CA and integral-CA, leading to a risk of incorrectly rejecting levofloxacin dosing regimens via midpoint-CA; i.e. not offering an adequate therapy.

Employing this knowledge will enable more accurate predictions of PK variabilities in the clinic, which will ultimately improve the understanding of drug target-site PK, in particular distribution into the target site, for therapeutic decision-making.

### ACKNOWLEDGEMENTS AND DISCLOSURES

Dr. Jens Borghardt and Francis Ojara are gratefully acknowledged for helpful discussions. The authors thank the High-Performance Computing Service at Freie Universitaet Berlin (http://www.zedat.fu-berlin.de/Compute) for providing high-performance computing capacities enabling our modelling and simulation activities. CK and WH report grants from an industry consortium (AbbVie Deutschland GmbH & Co. KG, AstraZeneca GmbH, Boehringer Ingelheim Pharma GmbH & Co. KG, Grünenthal GmbH, F. Hoffmann-La Roche Ltd., Merck KGaA and SANOFI) for the PharMetrX program. CK reports grants for the Innovative Medicines Initiative-Joint Undertaking (“DDMoRe”), Diurnal Ltd., the Federal Ministry of Education and Research within the Joint Programming Initiative on Antimicrobial Resistance Initiative (JPIAMR) and from the European Commission within in the Horizon 2020 framework programme (“FAIR”), all outside the submitted work. The other authors declare that they have no competing interests. Approval was obtained from the ethics committee of the University of Vienna. The procedures used in this study adhere to the tenets of the Declaration of Helsinki. Written informed consent was obtained from all individual participants included in the study. The datasets may be provided from MZ upon request. Model codes may be provided from CK upon request.

## Supplementary Information

ESM 1 (PDF 1.92 mb)

## ABBREVIATIONS

AUC_0–8_: Area under the concentration-time curve from 0 to 8 h
CAP: Community-acquired pneumonia
CFR: Cumulative fraction of response
CLCR: Creatinine clearance
C_max_: Maximum concentration
ECOFF: Epidemiological cut-off value
EUCAST: European Committee on Antimicrobial Susceptibility Testing
*f*AUC_0–8_: Area under the unbound concentration-time curve from 0 to 8 h
*f*AUC_0–24_: Area under the unbound concentration-time curve from 0 to 24 h
Integral-CA: Dialysate-based integral compartmental analysis
ISF: Interstitial space fluid
MIC: Minimum inhibitory concentration
Midpoint-CA: Dialysate-corrected mid-interval compartmental analysis
NCA: Noncompartmental analysis
NLME: Nonlinear mixed-effects
PD: Pharmacodynamic(s)
PK: Pharmacokinetic(s)
PTA: Probability of target attainment
RSE: Relative standard error
RR: Relative recovery
V_1_: Central volume of distribution
V_tot_: Total volume of distribution
