# Supplementary material for: Which Analysis Approach Is Adequate to Leverage Clinical Microdialysis Data? A Quantitative Comparison to Investigate Exposure and Response Exemplified by Levofloxacin
Source: Pharm Res. 2021 Mar 15;38(3):381–95. doi: 10.1007/s11095-021-02994-1 (PMC7994214; doi:10.1007/s11095-021-02994-1)
Supplement: Supplementary file 1 — (PDF 1.92 mb) [file 11095_2021_2994_MOESM1_ESM.pdf]

Stochastic and covariate submodels

For all investigated models, a log-normal distribution of individual pharmacokinetic (PK) model parameters was assumed and implemented as an exponential function according to Eq. S1:

$$P_{k,i} = \theta_k e^{\eta_{ki}} \qquad \text{(Eq. S1)}$$

where $P_{k,i}$ denotes the estimated value of the PK parameter k for the individual patient i, θ_k the typical value for PK parameter and η_{ki} the natural logarithmic difference between $P_{k,i}$ and θ_k.

Categorical covariates were implemented as proportional deviation from the reference parameter ($\theta_{categorical\ cov.}$):

$$P_{k,i} = \begin{cases} \theta_k \cdot e^{\eta\ k,i} \\ \theta_k \cdot \theta_{categorical\ cov.} \cdot e^{\eta\ k,i} \end{cases} \qquad \text{(Eq. S2)}$$

Continuous covariates were implemented as proportional change from the reference parameter ($\theta_{continuous\ cov.}$) with individual covariate values (cov_i) centred on the median covariate value (cov_{median}):

$$P_{k,i} = \theta_k \cdot (1 + \theta_{continuous\ cov.}\ (cov_i - cov_{median}) \cdot e^{\eta_{ki}} \qquad \text{(Eq. S3)}$$

Body size descriptors were implemented via allometric scaling with exponent θ_{BSD} fixed to either 1 for volumes or 0.75 for intercompartmental flows:

$$P_{k,i} = \theta_k \cdot \left(\frac{cov_i}{cov_{median}}\right)^{\theta_{BSD}} \cdot e^{\eta_{ki}} \qquad \text{(Eq. S4)}$$

The difference between individual model-predicted plasma concentrations ($Y_{IPRED,i,j}$) and observed data ($Y_{i,j}$) for each individual i at time-point j is depicted as residual variability ($\varepsilon_{i,j}$) using a proportional model (Eq. S5, other residual variability models were tested).

$$Y_{i,j} = Y_{IPRED,i,j} \cdot (1 + \varepsilon_{i,j}) \qquad \text{(Eq. S5)}$$

For the integration of micro- and retrodialysis data in the integrated dialysate-based compartmental analysis (integral-CA), sampling interval time as well as the concentration of

the dialysate effect compartment in the respective microdialysis catheter were recognised in the dataset (Eq. S6) and finally incorporated via Eq. S5.

The integral-CA was applied in the present work as [1,3]:

$$C_{\mu D_{[t_j, t_{j+1}]}} = \int_{t_j}^{t_{j+1}} C_{ISF}(t) \cdot RR \, dt \, / t_{j+1} - t_j \quad \text{(Eq. S6)}$$

The microdialysate concentration ($C_{\mu D[t_j,\ t_{j+1}]}, j \in \{1, \ldots, n-1\}$) was defined by the integral of the ISF concentration-time profile ($C_{ISF}(t)$) multiplied by the relative recovery value (RR) over the collection interval (T_{int} = $[t_j, t_{j+1}]$), which was then divided by the duration of the microdialysate sample collection ($t_{j+1} - t_j$).

Table AI. Population parameter estimates of the final levofloxacin plasma nonlinear mixed-effects model.

Model Parameter	Units	Plasma (RSE, %)
V_1	[L]	21.7 (9.5)
Q_1	[L/h]	60.3 (9.7)
V_2	[L]	64.3 (7.3)
CL	[L/h]	6.83 (8.3)
**Interindividual variability**		
on V_1	[%CV]	51.8 (22)
on V_2	[%CV]	42.3 (36)
on CL	[%CV]	49.3 (22)
**Residual variability**		
all measurements	[%CV]	7.80-9.78 (19-39)*

* Consideration of interstudy variability (expressed as proportional change to the study in healthy volunteers

CL: clearance, CV: coefficient of variation, Q_1: intercompartmental flow between plasma and peripheral compartment, RSE: relative standard error, V_1: central volume of distribution, V_2: peripheral volume of distribution.

Table AII. Parameter estimates of the final dialysate-corrected mid-interval compartmental analysis (midpoint-CA) and dialysate-based integral compartmental analysis (integral-CA).

**Model Parameter**	**Units**	**Midpoint-CA (95% CI)**	**Integral-CA (95% CI)**
V_1	[l]	4.82 (1.06-10.1)	15.5 (12.0-19.5)
Q_1	[l/h]	78.5 (52.7-110)	43.4 (18.2-57.3)
V_2	[l]	39.6 (28.6-54.8)	33.4 (16.0-47.7)
Q_2	[l/h]	25.5 (3.53-37.9)	42.6 (16.7-72.0)
V_3	[l]	16.9 (2.22-31.2)	16.2 (6.16-31.2)
Q_3	[l/h]	10.5 (6.38-25.5)	11.5 (5.75-31.5)
V_4	[l]	21.4 (9.20-42.2)	16.0 (6.59-39.4)
CL	[l/h]	7.35 (5.51-8.76)	7.78 (7.16-8.38)
Q_2-alb	$[(g/l)^{-1}]$	8.19% (0.122%-8.46%)	7.25% (0.849%-8.70%)
CL-CLCR	$[(ml/min)^{-1}]$	1.26% (0.675%-2.05%)	1.12% (0.837%-1.59%)
CL_{sepsis}	-	-52.8% (8.10%-73.6%)	-43.4% (17.9%-65.2%)
RR_{adi}	-	-	19.3% (16.3%-22.8%)
RR_{mus}	-	-	26.9% (22.2%-31.6%)
**Interindividual variability**			
on V_1	[%CV]	143 (89.3-238)	56.2 (31.1-72.3)
on V_2	[%CV]	51.2 (31.6-72.6)	65.0 (35.5-100)
on Q_2	[%CV]	-	72.6 (36.5-112)
on V_3	[%CV]	95.8 (52.7-146)	60.4 (6.13-88.1)
on V_4	[%CV]	92.8 (62.2-119)	71.7 (41.1-101)
on CL	[%CV]	46.5 (23.2-77.3)	26.4 (16.6-36.2)
on RR_{adi}	[%CV]	-	50.3 (38.3-61.1)
on RR_{mus}	[%CV]	-	41.0 (29.1-52.3)
**Residual variability**			
all measurements	[%CV]	30.3 (26.6-34.3)	-

μD_{adi}	[%CV]	-	28.3 (21.6-33.6)
μD_{mus}	[%CV]	-	25.4 (20.1-31.2)
RR_{adi}	[%CV]	-	18.4 (12.7-23.2)
RR_{mus}	[%CV]	-	24.9 (19.0-32.0)
Plasma	[%CV]	-	8.83 (7.54-10.2)

Adi: adipose tissue, CL: clearance, CLCR: creatinine clearance, CL-CLCR: relationship between CL and CLCR, CV: coefficient of variation, mus: muscle, Q_1: intercompartmental flow between plasma and unspecified compartment, Q_2: intercompartmental flow between plasma and adipose tissue, Q_2-alb: relationship between Q_2 and serum albumin concentration, Q_3: intercompartmental CL between plasma and muscle tissue, V_1: central volume of distribution, V_2: unspecified peripheral volume of distribution, V_3: adipose associated volume of distribution, V_4: muscle associated volume of distribution, RR_{adi}: relative recovery adipose tissue, RR_{mus}: relative recovery muscle, µD: microdialysis.

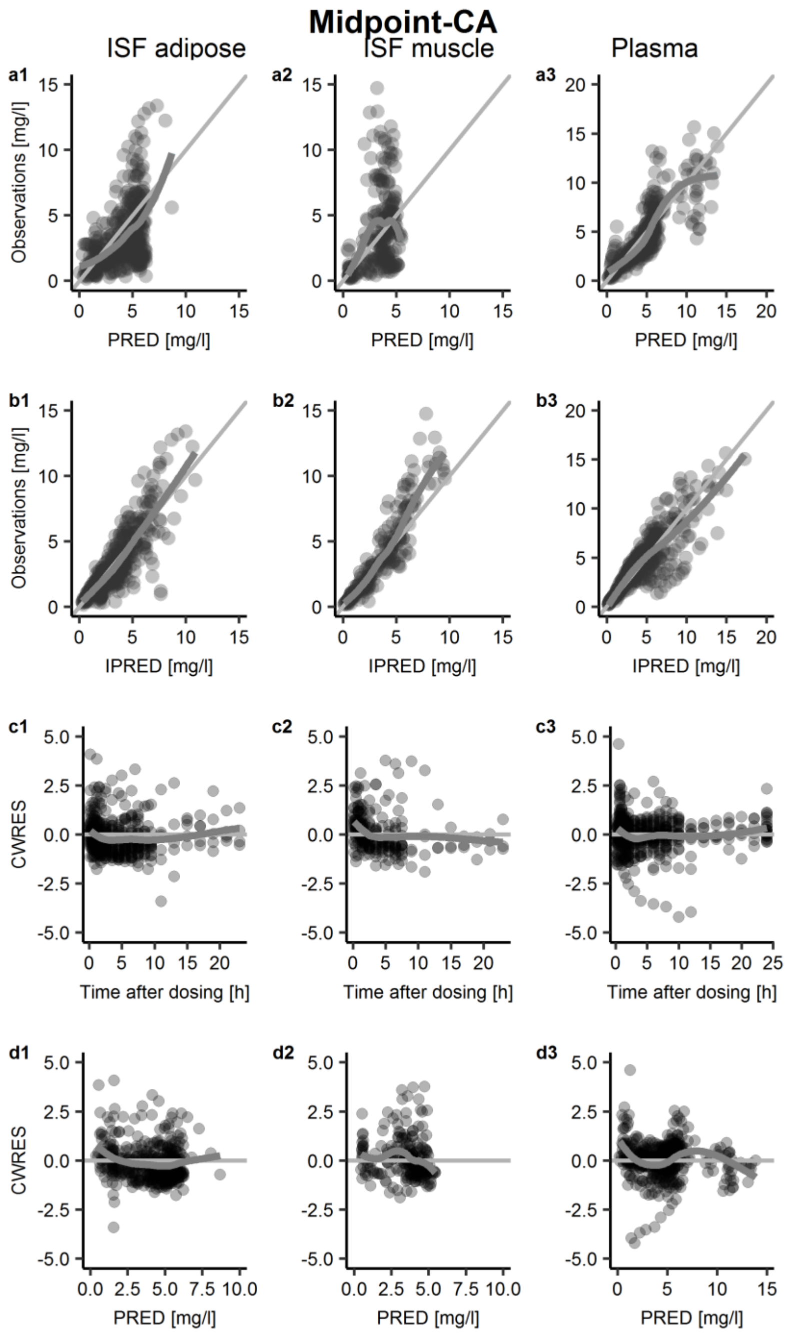

Figure A1. Basic goodness-of-fit (GOF) plots for the final dialysate-corrected mid-interval compartmental analysis (midpoint-CA). Rows present GOF plots of observations versus population predictions (PRED, a) observations versus individual predictions (IPRED, b), conditional weighted residuals (CWRES) versus time after dosing (c) and CWRES versus PRED (d). Columns show GOF plots for levofloxacin concentrations in the intersititial space fluid (ISF) in adipose tissue (1), muscle (2) and plasma (3). Straight line represents unity line, bold grey line represents loess smoother.

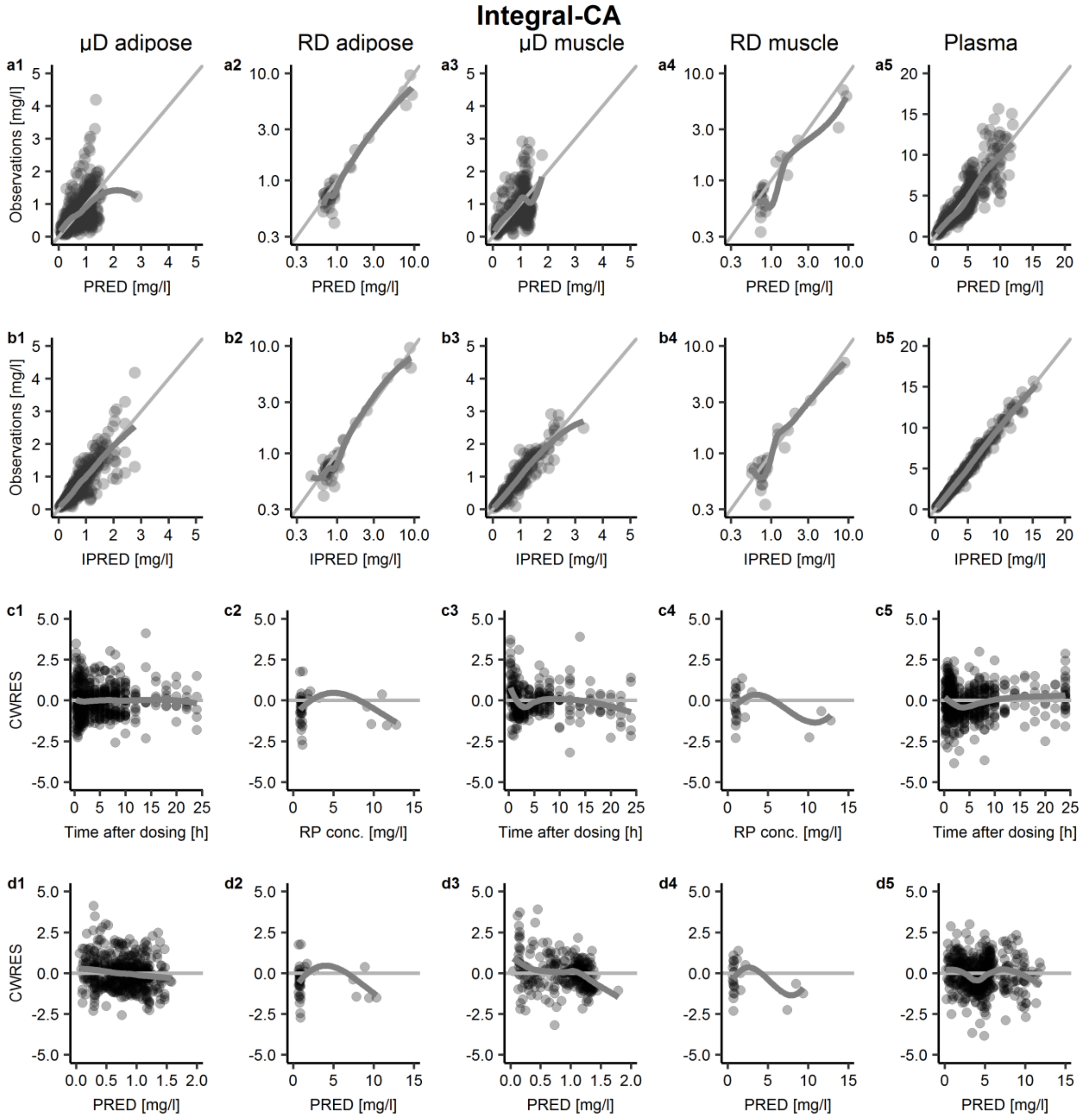

Figure A2. Basic goodness-of-fit (GOF) plots for the final dialysate-based integral compartmental analysis (integral-CA). Rows present GOF plots of observations versus population predictions (PRED, a) observations versus individual predictions (IPRED, b), conditional weighted residuals (CWRES) versus time after dosing (c) and CWRES versus PRED (d). Columns show GOF plots for levofloxacin microdialysate concentrations (µD) and (1) retrodialysate concentrations (RD) in adipose tissue (2), µD in muscle (3) and RD in muscle (4) and plasma concentrations (5). Straight line represents unity line, bold grey line represents loess smoother.

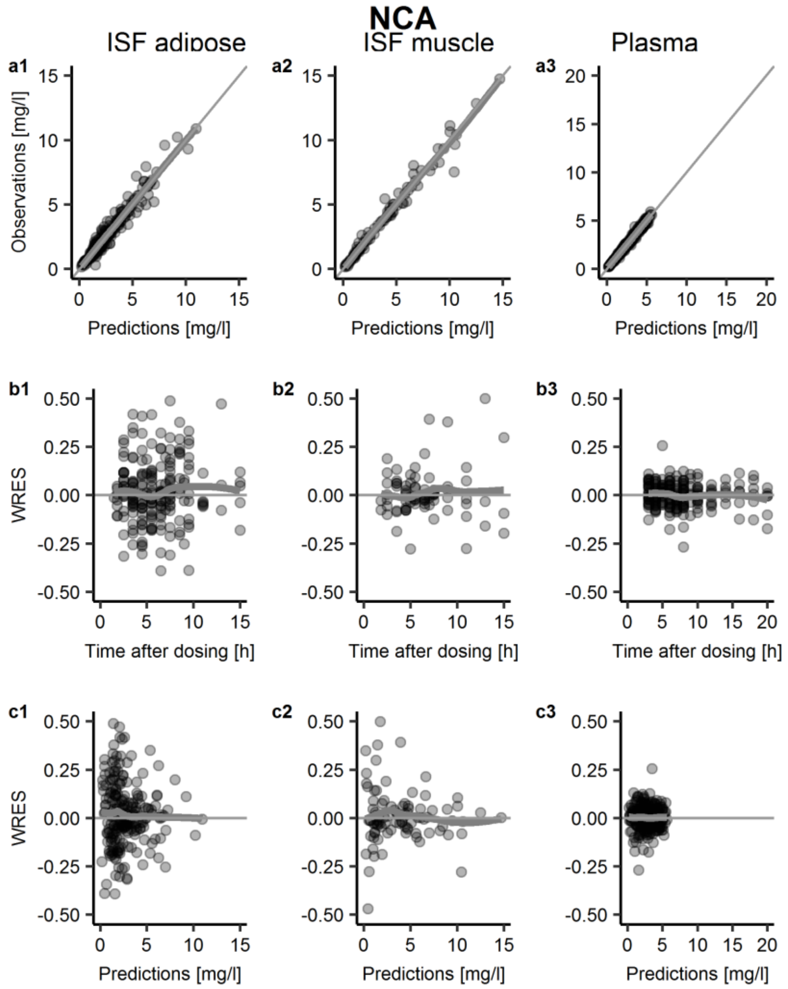

Figure A3. Basic goodness-of-fit (GOF) plots for the loglinear regression of the terminal phase levofloxacin concentrations versus time via noncompartmental analysis (NCA). Rows present GOF plots of observations versus predictions (a), weighted residuals (WRES) versus time after dosing (b) and WRES versus predictions (c). Columns show GOF plots for levofloxacin concentrations in the intersititial space fluid (ISF) in adipose tissue (1), muscle (2) and plasma (3). Straight line represents unity line, bold grey line represents loess smoother.

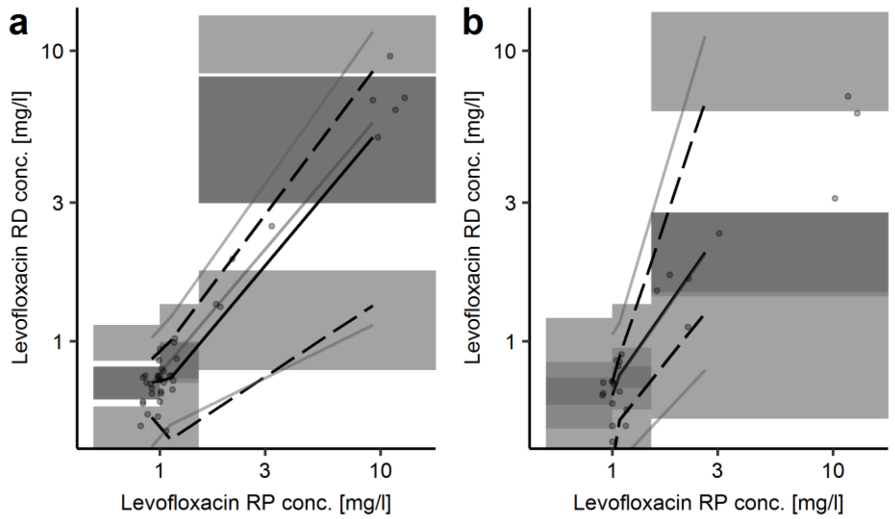

Figure A4. Visual predictive check (n=1000 simulations) for the dialysate-based integral compartmental analysis for retrodialysate concentrations for adipose tissue (a) and muscle (b). Circles: Observed levofloxacin concentrations; Lines: 5th, 95th percentile (dashed), 50th percentile (solid) of the observed (black) and simulated (grey) data. Shaded areas: 95% confidence interval around 5th, 50th and 95th percentile of simulated data. RD: retrodialysate; RP: retroperfusate.

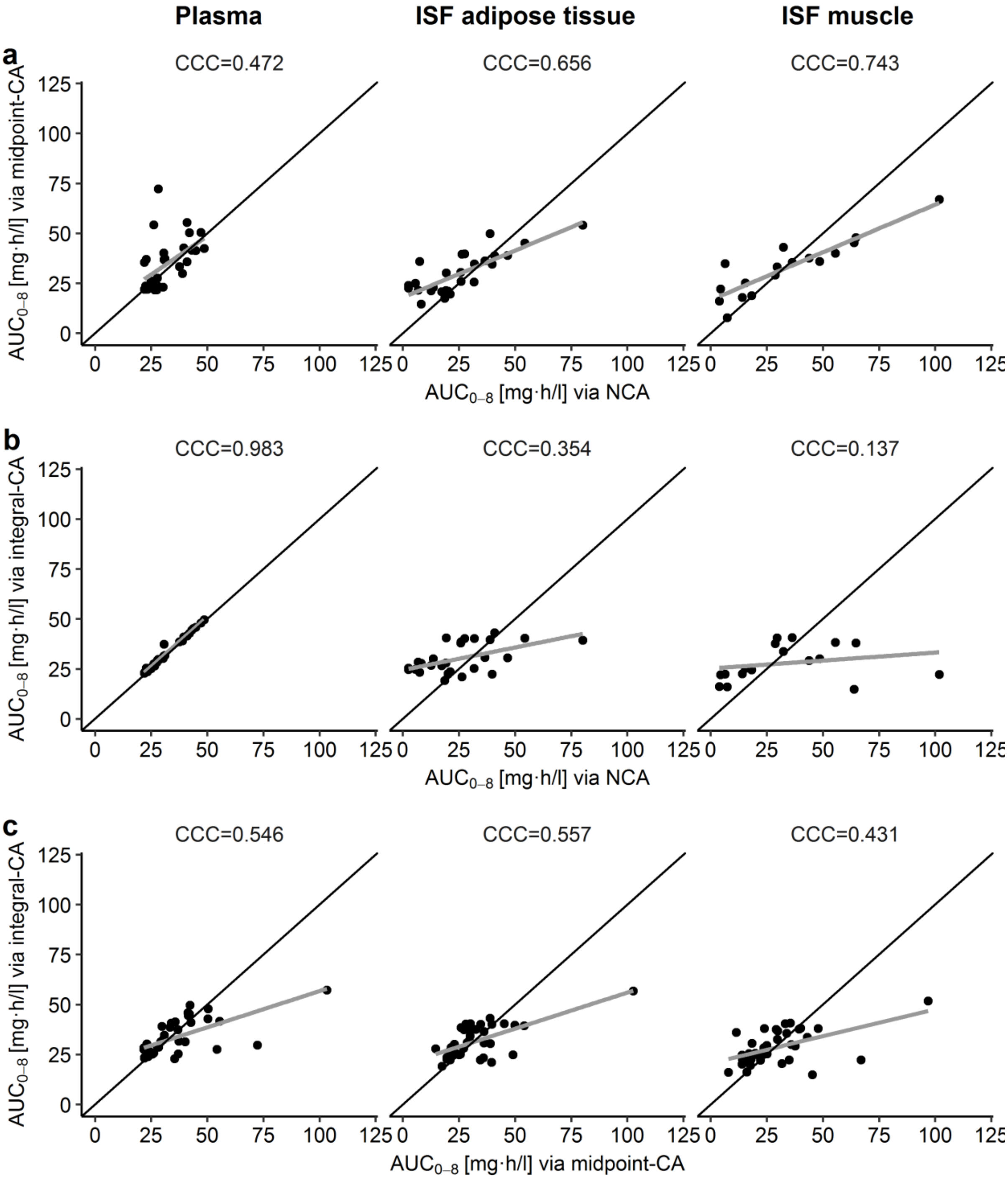

Figure A5. Comparison of individual area under the concentration-time curves from 0-8 h ($AUC_{0\text{-}8}$) determined via noncompartmental analysis (NCA), dialysate-corrected mid-interval compartmental analysis (midpoint-CA) and dialysate-based integral compartmental analysis (integral-CA). Rows show separate comparisons of analysis appraoches and columns show sampling matrices. Black line represents the unity line, grey lines represent linear regression lines. CCC: Lin's concordance correlation coefficient, ISF: interstitial space fluid.

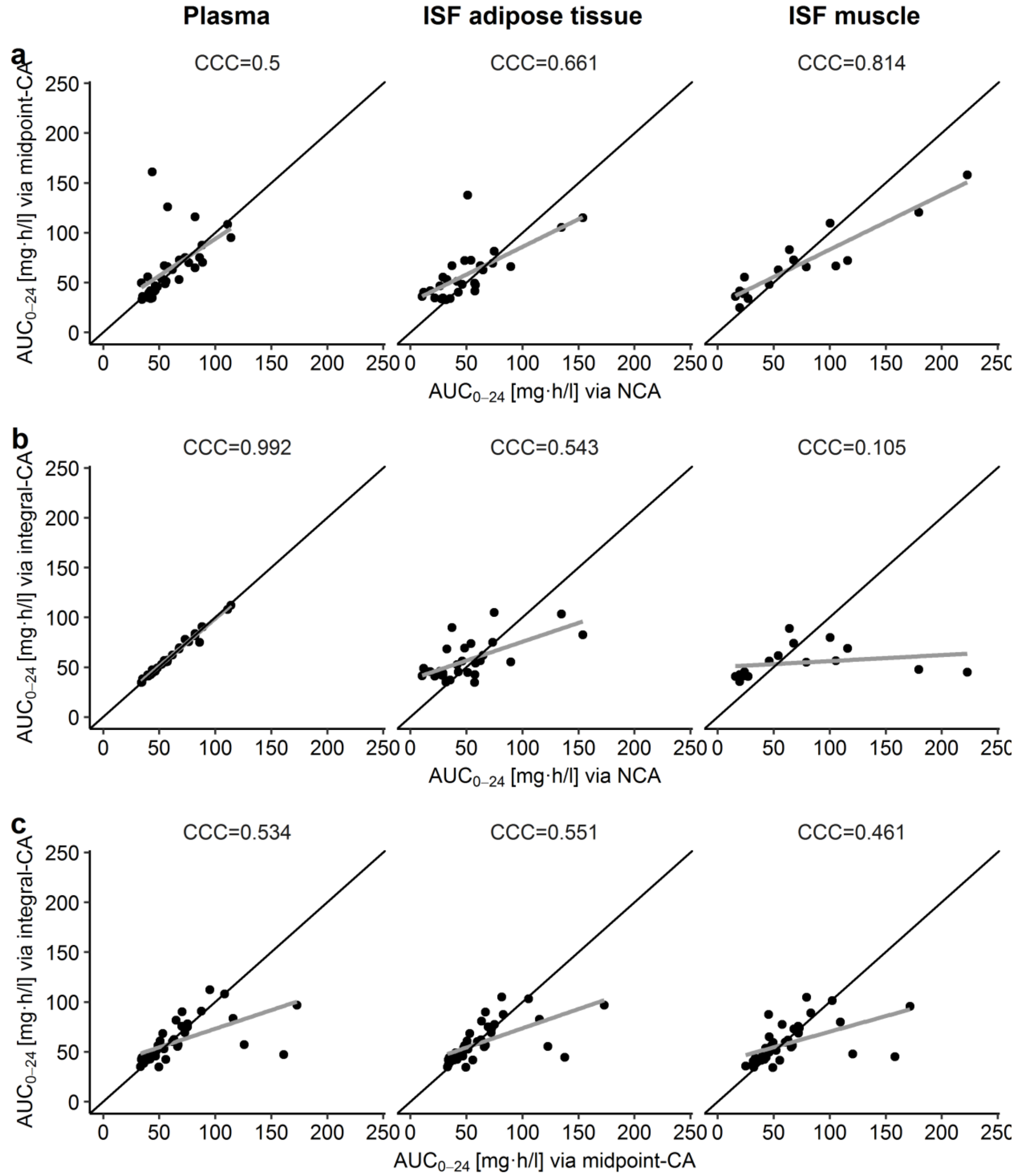

Figure A6. Comparison of individual area under the concentration-time curves from 0-24 h ($AUC_{0\text{-}24}$) determined via noncompartmental analysis (NCA), dialysate-corrected mid-interval compartmental analysis (midpoint-CA) and dialysate-based integral compartmental analysis (integral-CA). Rows show separate comparisons of analysis appraoches and columns show sampling matrices. Black line represents the unity line, grey lines represent linear regression lines. CCC: Lin's concordance correlation coefficient, ISF: interstitial space fluid.

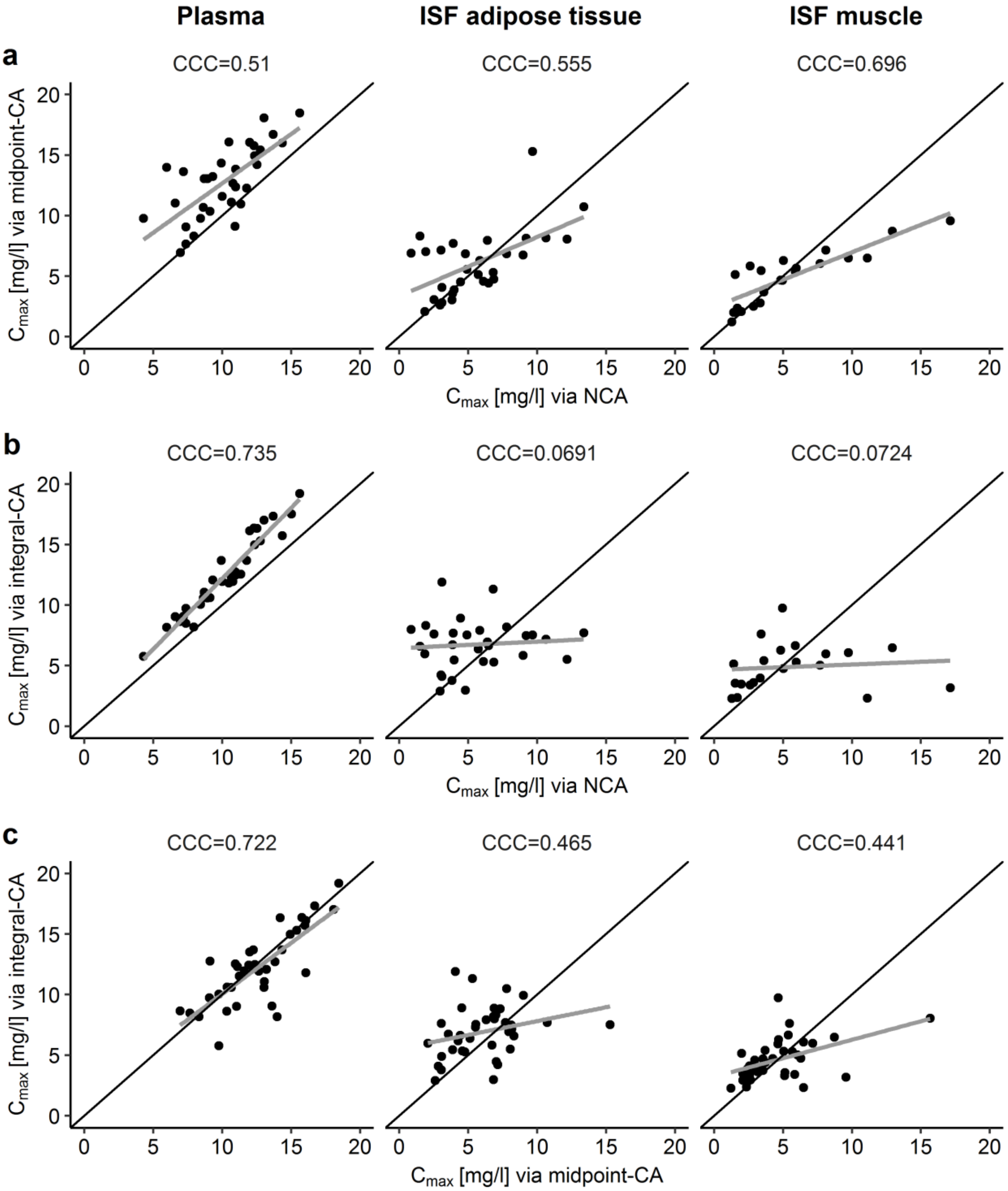

Figure A7. Comparison of individual maximum concentration (C_{max}) determined via noncompartmental analysis (NCA), dialysate-corrected mid-interval compartmental analysis (midpoint-CA) and dialysate-based integral compartmental analysis (integral-CA). Rows show separate comparisons of analysis appraoches and columns show sampling matrices. Black line represents the unity line, grey lines represent linear regression lines. CCC: Lin's concordance correlation coefficient, ISF: interstitial space fluid.

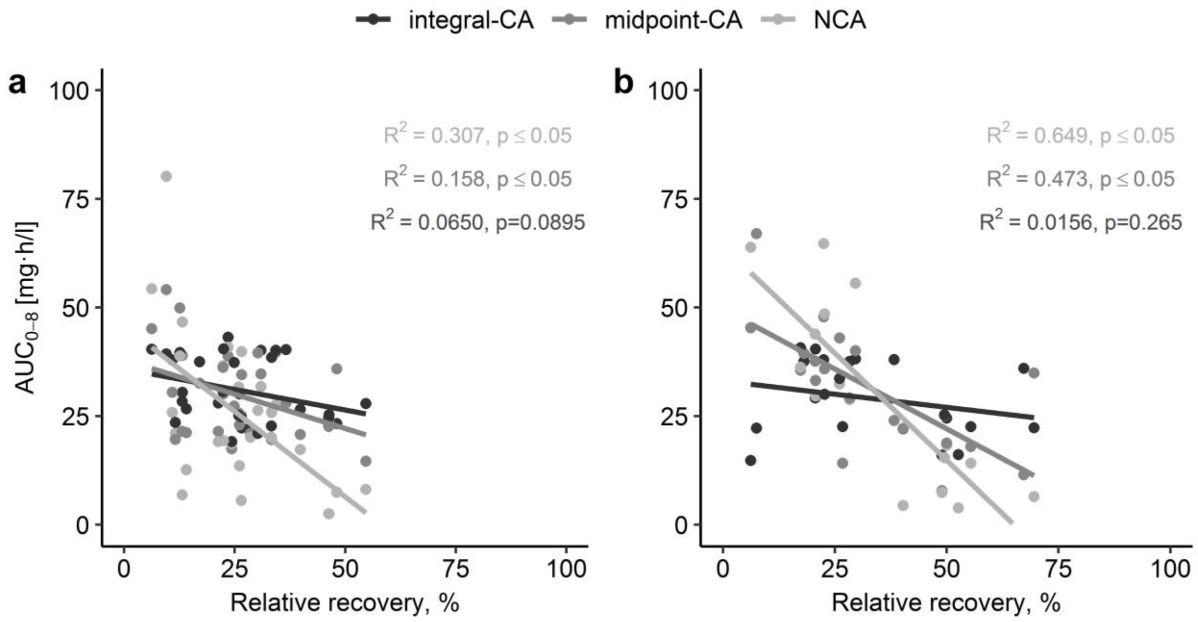

Figure A8. Individual area under the concentration-time curves from 0-8 h ($AUC_{0\text{-}8}$) in interstitial space fluid of adipose tissue (a) and muscle (b) determined via noncompartmental analysis (NCA), dialysate-corrected mid-interval compartmental analysis (midpoint-CA) and dialysate-based integral compartmental analysis (integral-CA) versus relative recovery in the same matrices. Grey lines represent linear regression lines.
